# Proteostasis Environment Shapes Higher-Order Epistasis Operating on Antibiotic Resistance

**DOI:** 10.1534/genetics.119.302138

**Published:** 2019-04-23

**Authors:** Rafael F. Guerrero, Samuel V. Scarpino, João V. Rodrigues, Daniel L. Hartl, C. Brandon Ogbunugafor

**Affiliations:** * Department of Computer Science, Indiana University, Bloomington, Indiana 47408; † Network Science Institute, Northeastern University, Boston, Massachusetts 02115; ‡ Department of Marine and Environmental Sciences, Northeastern University, Boston, Massachusetts 02115; § Department of Physics, Northeastern University, Boston, Massachusetts 02115; ** Department of Chemistry and Chemical Biology, Harvard University, Cambridge, Massachusetts 02138; †† Department of Organismic and Evolutionary Biology, Harvard University, Cambridge, Massachusetts 02138; §§ Department of Ecology and Evolutionary Biology, Brown University, Providence, Rhode Island 02912

**Keywords:** epistasis, proteostasis, antibiotic resistance

## Abstract

Recent studies have affirmed that higher-order epistasis is ubiquitous and can have large effects on complex traits. Yet, we lack frameworks for understanding how epistatic interactions are influenced by central features of cell physiology. In this study, we assess how protein quality control machinery—a critical component of cell physiology—affects epistasis for different traits related to bacterial resistance to antibiotics. Specifically, we disentangle the interactions between different protein quality control genetic backgrounds and two sets of mutations: (i) SNPs associated with resistance to antibiotics in an essential bacterial enzyme (dihydrofolate reductase, or DHFR) and (ii) differing DHFR bacterial species-specific amino acid background sequences (*Escherichia coli*, *Listeria grayi*, and *Chlamydia muridarum*). In doing so, we improve on generic observations that epistasis is widespread by discussing how patterns of epistasis can be partly explained by specific interactions between mutations in an essential enzyme and genes associated with the proteostasis environment. These findings speak to the role of environmental and genotypic context in modulating higher-order epistasis, with direct implications for evolutionary theory, genetic modification technology, and efforts to manage antimicrobial resistance.

## Abstract (precis)

Epistasis is widely regarded as one of the most important phenomena in genetics. It proposes that the combined effects of mutations cannot be easily predicted from their individual effects. In the present study...

INTERACTIONS between the different sources and levels of genetic information (*e.g.*, mutations, gene variants, and gene networks), as captured in phenomena like pleiotropy and epistasis, are widely recognized as a powerful force in crafting the relationship between genotype and phenotype (Cordell 2002; Remold and Lenski 2004; Phillips 2008; Natarajan *et al.* 2013; Chou *et al.* 2014; Mackay and Moore 2014; Sackton and Hartl 2016; Crona *et al.* 2017; Otwinowski *et al.* 2018). Epistasis—informally defined as the “the surprise at the phenotype when mutations are combined, given the constituent mutations’ individual effects” (Weinreich *et al.* 2013)—is now a highly relevant frontier of evolutionary genetics. It casts a shadow over many areas of biology that aim to understand or manipulate genetic variation [*e.g.*, genome-wide association studies (GWAS) and genetic modification), as it speaks to unpredictability regarding how phenotypes are related to the genes that are presumed to underlie them.

An especially provocative related phenomenon is “higher-order epistasis.” It offers that units of genetic information not only interact in a pairwise fashion (*e.g.*, mutation A interacting nonlinearly with mutation B and mutation B interacting nonlinearly with mutation C) but potentially in all possible combinations, each with a potentially unique statistical effect (*e.g.*, when the interaction between all three mutations––A, B, and C––has a quantitative value that cannot be reduced to a combination of independent or pairwise effects) (Weinreich *et al.* 2013; Poelwijk *et al.* 2016; Crona *et al.* 2017; Sailer and Harms 2017). Statistically, higher-order epistasis is an unwieldy concept because the number of possible interactions can grow exponentially with the number of interacting entities, which presents both conceptual and computational challenges (as it is a mental challenge to keep track of thousands of potential interactions, and computationally challenging to analyze them using available technology).

Many studies of higher-order epistasis focus on the interactions between suites of SNPs associated with a certain phenotype, engineered in combination or via a library of mutations using high-throughput methods (Ferretti *et al.* 2016; Poelwijk *et al.* 2016; Crona *et al.* 2017; Domingo *et al.* 2018; Li and Zhang 2018; Otwinowski *et al.* 2018; Tamer *et al.* 2018). Fewer studies specifically dissect the strength and sign of epistatic interactions between SNPs within a gene, and particular suites of mutations or gene deletions in other parts of the genome (Williams *et al.* 2005; Lehner 2011; Vogwill *et al.* 2016). Even fewer dissect the impact of physiological contexts on epistasis, a glaring omission when you consider the biochemical and biophysical specifics of the cellular environment, in which genes and proteins are made, and function.

One particular context that we might predict would shape epistasis within a cell would be that dictated by sets of chaperones and proteases, which have already been demonstrated to impact a range of bacterial phenotypes (Gottesman *et al.* 1997; Tokuriki and Tawfik 2009). Prior studies focusing on the chaperonins GroEL/ES and Lon protease have established their centrality in regulating the presence and state of only certain proteins in the cytoplasm (Hartl *et al.* 2011). And even more recent studies have uncovered how only members of this protein quality control (PQC) system (GroEL/ES and Lon) specifically stabilize different variants of dihydrofolate reductase (DHFR) (Bershtein *et al.* 2013). The allelic resolution of this proteostasis machinery is a striking finding, and begs the question of how this machinery might frame higher-order epistasis in traits that are controlled by specific proteins.

Here, we quantify the magnitude, sign, and order of epistatic effects acting on three mutations within a gene, as influenced by three well-defined proteostasis environments (conferred through the engineering of three genotypes of bacteria): wild-type, GroEL+ (overexpression), and Δ*lon*. We examine these effects for two related traits that contribute to antibiotic resistance (${\text{IC}}_{\text{50}}$ and DHFR abundance) (Rodrigues *et al.* 2016), and decompose the impact of the proteostasis environment on two classes of potential epistatic interactors: (i) three biallelic sites associated with drug resistance in an enzyme target of antibiotics (DHFR) and (ii) three different amino acid backgrounds corresponding to species of bacteria (*Escherichia coli*, *Chlamydia muridarum*, and *Listeria grayi*). We find that the sign and magnitude of interactions among SNPs is highly contingent upon certain genotypic contexts, and observe that epistasis can work differently (qualitatively and quantitatively) across related traits. Importantly, because the biology of the system under study is well understood (*e.g.*, the biophysics of variation in DHFR function, the basis through which the PQC machinery regulates proteins), we can surmise on the mechanism underlying certain epistatic interactions at work in the study system. We discuss these findings in light of theory in evolutionary genetics, the study of antibiotic resistance, and the challenges facing genetic modification technology.

## Materials and Methods

### Strains and phenotypes

Our collection of strains, which are a subset of those originally engineered for the study of DHFR structure and function by Bershtein *et al.* (2013), includes mutants from three species: *E. coli* (accession: P0ABQ4), *L. grayi* (accession: WP 003758501), and *C. muridarum* (accession: WP 010231888). We measured phenotypic effects of mutations at three sites in the FolA gene encoding DHFR (which we denote $DHFR_{Ec}$, $DHFR_{Lg}$, and $DHFR_{Cm}$, corresponding to species *E. coli*, *L. grayi*, and *C. muridarum*, respectively). We encoded the allelic state of a strain using binary notation, 000 corresponding to the ancestor (containing no mutations) and 111 containing all three focal mutations, as is common in these types of combinatorial data sets. For simplicity, we refer to individual sites by their position and amino acid change in $DHFR_{Ec}$ (even though these can be different in the other two species; see below).

We initially chose ${\text{IC}}_{\text{50}}$, protein abundance, and drugless growth rate as traits of interest. ${\text{IC}}_{\text{50}}$, a proxy for the ability of an organism to withstand the activity of antibiotics (trimethoprim in this case), is largely determined by several factors, including abundance and drugless growth rate (Rodrigues *et al.* 2016).

#### Construction of the PQC mutants:

Genes encoding ATP-dependent protease Lon were deleted using homologous recombination enhanced by λ red, essentially as described previously (Datsenko and Wanner 2000). Wild-type *E. coli* K12 MG1655 cells were cotransformed with various pFLAG-DHFR mutants and pGro7 plasmid (Takara) expressing groES-groEL under the pBAD promoter. Chaperone expression was induced by the addition of 0.2% arabinose.

#### Construction of the DHFR mutants:

Combinatorially complete sets of mutants were constructed for all three species orthologs of DHFR, for the three sites of interest in the FolA gene ($DHFR_{Ec}$, P21L, A26T, and L28R; $DHFR_{Cm}$, P23L, E28T, and L30L; and $DHFR_{Lg}$, P21L, A26T, and L28R). These mutations were introduced using a Quick-Change Site-Directed Mutagenesis Kit (Stratagene, La Jolla, CA) and cloned into the pFLAG expression vector (Sigma [Sigma Chemical], St. Louis, MO). Each mutagenized plasmid underwent confirmatory sequencing.

#### Measurement of ${\text{IC}}_{\text{50}}$:

As with the drugless growth rate, bacteria were grown across a range of concentrations of trimethoprim ranging from 0 to 2500 μg/ml) and incubated at 37°. Absorbance measurements at 600 nm were taken every 30 min for 15 hr. OD readings *vs.* time were calculated between 0 and 15 hr. ${\text{IC}}_{\text{50}}$ values were determined from the fit of a logistic equation to plots of growth *vs.* trimethoprim concentrations. Reported ${\text{IC}}_{\text{50}}$ are averaged from at least three replicates. To obtain the ${\text{IC}}_{\text{50}}$ results, growth measurements were conducted at the following trimethoprim concentrations (microgram per milliliter): 2500, 500, 100, 20, 4, 0.8, 0.16, 0.032, 0.0064, 0.00128, and 0.

#### Measurements of intracellular protein abundance:

DHFR abundance was measured from the total catalytic activity of the varying alleles in cellular lysates using methods similar to those outlined in a prior study (Rodrigues *et al.* 2016). Overnight cultures grown at 37° in M9 minimal medium supplemented with 2 g/liter glucose and 100 mg/liter ampicillin were diluted in fresh medium to an OD of 0.1 (final volume of 1 ml). At this point, arabinose (0.2% final concentration) was added to cultures of cells harboring GroEL/ES-expressing plasmid. After 5–6 hr, the ODs of the cultures were recorded, the cells were pelleted by centrifugation, and then lysed by the addition of 100 μl 1× Popculture reagent (Millipore, Bedford, MA), 1× Complete protease inhibitor cocktail (Roche), and 1 mM dithiothreitol. After 20 min incubation at room temperature with shaking, the lysates were cleared by centrifugation and the soluble fractions transferred to a 96-well plate for total enzymatic activity determination. Different volumes of cell lysates were preincubated with 100 μM NADPH and the reaction was started by adding 50 μM dihydrofolate. The reaction was followed by fluorescence (excitation at 300 nm and emission at 400 nm) and the initial slopes were computed. Enzyme concentration in lysates was determined by dividing the total enzymatic activity by ${\text{k}}_{\text{cat}}$, which in turn was converted to DHFR molecules/cell taking into consideration the measured OD and that 1 ml of cells at OD = 1.0 has ∼$10^{9}$ cells.

Note regarding protein abundance: how much DHFR is produced by a given cell is the product of many biochemical and biophysical actors. DHFR abundance is an important component of drug resistance, because to survive the presence of trimethoprim (which disrupts the biosynthesis of a folate, a key metabolite; see Supplemental Material, Supplemental Information), the organism must produce enough DHFR to carry out normal cellular function. Also, because we know that PQC machinery, like GroEL and Lon protease, can degrade proteins like DHFR (Bershtein *et al.* 2013; Rodrigues *et al.* 2016), there is a physiological basis for an expectation that these PQC genetic backgrounds would influence protein abundance.

### Statistical analysis

Our approach, an application of regularized regression techniques, allows us to measure higher-order epistasis acting across traits and biological scales (*e.g.*, within and between genes). These methods can be used to infer statistical interactions operating in experimental and natural data sets. This regression approach can be applied to data sets of varying structure, can easily incorporate experimental noise, and can produce results for data sets with missing values (even though the data set in this study is combinatorially complete). The limits of regression methods have been explored in other studies of epistasis (Otwinowski and Plotkin 2014; Sailer and Harms 2018); however, in the Supplemental Information, we demonstrate that the regularized regression methods utilized here are consistent with other methods, such as those that explore “global” epistasis (Sailer and Harms 2017; Otwinowski *et al.* 2018).

#### Initial exploration:

We set out to infer interactions across three bacterial traits: ${\text{IC}}_{\text{50}}$, DHFR abundance, and bacterial growth rate (total experimental *N* = 232, 360, and 252, respectively). For each phenotype, we first fitted a general linear model of the form Y∼S+C+H, where *Y* is the phenotype of interest (${\text{IC}}_{\text{50}}$, abundance, or growth), *S* is the species fixed factor (with three levels), *C* is the PQC context (wild-type, Δ*lon*, and GroEL+), and *H* is a haplotype variable (with eight levels, coding for the possible combinations of mutations P21L, A26T, and L28R). We tested for the presence of epistasis by fitting alternative models that include the interaction terms S×C, S×H, C×H, and S×C×H, and choosing the model of best fit based on the Bayesian information criteria (*i.e.*, BIC; a penalty for added regression coefficients proportional to the natural log of the sample size), and a combination of forward and reverse model selection as implemented in the R programming language’s stats package (R Core Team 2018). After finding significant interaction effects in these initial models for ${\text{IC}}_{\text{50}}$ and protein abundance, we proceeded to carry out further analyses on these two phenotypes. The drugless growth rate data did not demonstrate evidence for higher-order epistasis using BIC (Figure S1), and so we did not carry out further analyses of the drugless growth rate.

#### Regularized regressions (Elastic Net/least absolute shrinkage and selection operator):

We tested for epistasis by fitting regularized regressions, which select the set of explanatory variables and estimate their coefficients in a single procedure. Briefly, this is done by including penalties proportional to the value of each coefficient (corresponding to each explanatory variable) in the regression equation. As with other regression procedures (*e.g.*, least-squares), the objective is to minimize this (penalized) equation. In doing so, it finds a balance between small coefficient values and errors in the fit of the model. If a variable does not affect the phenotype of interest, its coefficient will be zero. We took nonzero coefficients as evidence that a particular variable, or interaction term, has a significant effect on the phenotype.

We fitted these models on standardized phenotypic variables, allowing direct comparisons between the coefficients estimated from different regressions (*i.e.*, units for regression coefficients are SD). Prior to standardization, we log-transformed abundance and ${\text{IC}}_{\text{50}}$ values to improve normality, and ruled out a large effect of nonlinear genotype–phenotype relationships (see Supplemental Material). We ran the regression procedures using the glmnet package (Friedman *et al.* 2010) in R, which carries out an Elastic Net regularization. Specifically, we used the “cv.glmnet” method, which fits models with varying penalty weights (changing the λ parameter) and finds the best model by cross-validation (in our case, a leave-one-out approach). To avoid overfitting, we chose the simplest model that is still within one cross-validated SE of the best fit model (that is, using $\lambda_{min}+1\text{SE}$). The Elastic Net method combines linear and quadratic penalties (in α and 1 − α proportions, respectively) to obtain a sparse set of variables. In the *Results* section, we present regressions using α = 1, which yields fewer nonzero coefficients (which we deem to be more conservative) and is equivalent to a LASSO (least absolute shrinkage and selection operator) approach (Tibshirani 1996). Regressions using other values of α had little effect on the qualitative patterns (see Data S1 for both data sets: α = 1 and 0.5).

For each phenotype, we fitted models at two scales. First, we ran a full model (Z∼S×C×P21L×A26T×L28R) that included 72 terms: the main effects of five variables (species, PQC context, and the mutations P21L, A26T, and L28R) and all possible interactions. Second, we ran models within PQC-species context (that is, nine separate models per phenotype) to get a more detailed perspective on how PQC shapes intragenic epistatic interactions. Within each PQC-species context, the model fit was w∼P21L×A26T×L28R, where *w* is the phenotypic value normalized within each group (*i.e.*, using the mean and variance of each PQC-species set). The estimated coefficients for these models are summarized in Data S2).

### Data availability

All data and scripts for these analyses—written in R (R Core Team 2018) and using methods in the tidyverse (Wickham 2017), glmnet (Friedman *et al.* 2010), and treemapify (Wilkins 2018) packages—can be found at https://github.com/guerreror/dhfr. Supplemental data are as follows. Data S1 concerns epistastic decomposition: regression effect sizes by order for ${\text{IC}}_{\text{50}}$, protein abundance, and drugless growth, for α = 0.5 and 1.0. Data S2 outlines transgenic SNP analyses: these are the data displayed in Figure S2, which demonstrate the phenotypic effects of individual SNPs and SNP combinations. Data S3 concerns the biophysical properties of the mutants as measured in prior studies (Rodrigues *et al.* 2016). We supply them here because they are the basis for speculations on the mechanisms underlying some of the epistatic interactions measured in this study (as discussed in Table 1 and Table 2). Supplemental material available at FigShare: https://doi.org/10.25386/genetics.8026775.

**Table 1:** Possible mechanisms underlying the five largest factors affecting ${\text{IC}}_{\text{50}}$

Effect	Category	Magnitude	Mechanistic interpretation
$DHFR_{Cm}$	Species (main effect)	−1.44	The *C. muridarum* amino acid background is thermodynamically unstable, more prone to proteolytic degradation, and has low catalytic efficiency. Consequently, it has a strong negative effect on the ability to survive in the presence of drug, across all other interacting genetic backgrounds.
L28R	SNP (main effect)	+1.22	The L28R mutation greatly increases both structural stability and the drug inhibition constant $\left({\text{K}}_{\text{i}}\right)$, and, consequently, helps DHFR perform its enzymatic function in the presence of drug, across genotypic contexts.
$DHFR_{Lg}$	Species (main effect)	−0.90	The *L. grayi* amino acid background is very thermodynamically unstable and prone to proteolytic degradation. This is partially compensated for by reasonably high catalytic efficiency $\left(K_{cat}/{\text{K}}_{\text{m}}\right)$, but still has a net negative effect on ${\text{IC}}_{\text{50}}$.
$DHFR_{Lg}$: P21L	Species × SNP (second-order)	−0.82	The *C. muridarum* amino acid background is inefficient and thermodynamically unstable. However, the P21L mutation is slightly stabilizing, which diminishes the negative impact of the *C. muridarum* amino acid background. The net effect remains negative, however. This result highlights how powerful the *C. muridarum* amino acid background is, in that it can “drag down” the positive effects of certain SNPs.
$DHFR_{Lg}$:L28R	Species × SNP (second-order)	−0.69	This highlights the nonlinear interaction between a powerfully positive SNP (L28R) and the strongly negative main effect *L. grayi* background. That the interaction term is negative highlights that even the stabilizing effects of a positive effect SNP (L28R) cannot compensate for the negative effects of the unstable *L. grayi* amino acid background.

DHFR, dihydrofolate reductase.

**Table 2:** Possible mechanisms underlying the five largest factors affecting DHFR abundance

Effect	Category	Magnitude	Mechanistic interpretation
$DHFR_{Lg}$: A26T: L28R	Species × SNP × SNP (third-order)	+1.59	The strongly positive effect of this third-order interaction is emblematic of the restorative effects of A26T:L28R, even on backgrounds typified by low availability, as in *L. grayi* (effect size = −0.84).
$DHFR_{Cm}$	Species (main effect)	−1.01	As described in Table 1 (as applied to its effect on ${\text{IC}}_{\text{50}}$), the *C. muridarum* amino acid background has low functional availability and low catalytic efficiency. These factors contribute to its negative impact on both ${\text{IC}}_{\text{50}}$ and abundance.
$DHFR_{Lg}$:L28R	Species × SNP (second-order)	−1.01	The L28R mutation, in isolation, is associated with DHFR thermostability and, relatedly, abundance (effect size = 0.88). The *L. grayi* background has a net negative effect on abundance (effect size = −0.84). Therefore, one might predict that their combination might cancel out toward a nearly neutral effect. Instead, this interaction has a net negative effect on abundance, an example of how some effects cannot be easily interpreted from knowledge of the underlying biochemistry of the enzyme.
L28R	SNP (main effect)	+0.88	The L28R SNP has a strong positive effect on DHFR thermostability, which is at least partly correlated with protein abundance.
$DHFR_{Lg}$: GroEL+: A26T: L28R	Species × PQC × SNP × SNP (fourth-order)	+0.87	The interaction between the A26T:L28R double mutant and the *L. grayi* amino acid background has a strongly positive effect on abundance (effect size = 1.59) that is somehow diminished in the presence of the GroEL+ PQC background. This is peculiar when we consider the positive GroEL+ main effect (effect size = 0.25). This implies that the positive effect (in terms of magnitude and direction) of the GroEL+ PQC background is specific to the SNP and amino acid combinations present in DHFR, a finding for which there is no simple, intuitive explanation.

DHFR, dihydrofolate reductase.

## Results

**Figure 1:**
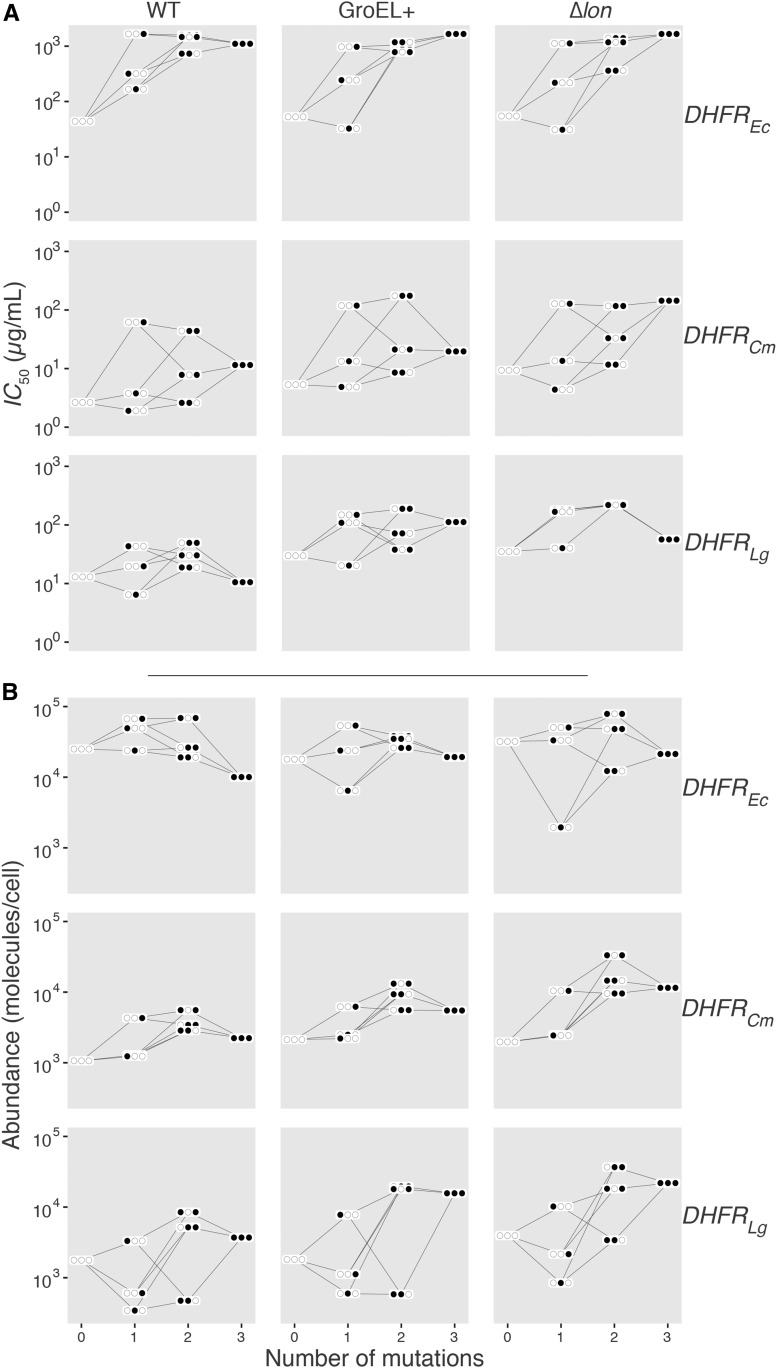
Phenotypic variation of DHFR mutants across proteostasis contexts. ${\text{IC}}_{\text{50}}$ (A) and abundance (B) depend on protein quality control context (panel rows) and species background (panel columns). DHFR mutations at three amino acid positions are represented by closed circles (first site = P21L, second site = A26T, and third site = L28T). DHFR, dihydrofolate reductase; WT, wild-type.

We first set out to construct a coarse picture of the experimental data: whole alleles of DHFR, with SNPs in various combinations engineered into several background strains, and assayed for three traits relevant to drug resistance. Figure 1 shows how the engineered alleles (the eight combinatorial mutants) perform with respect to ${\text{IC}}_{\text{50}}$ and protein abundance across genotypic contexts (species and PQC background). While these two phenotypes show patterns highly consistent with epistatic interactions at several levels, growth rate shows no significant variance across genotypic contexts (confirmed by Generalized Linear Models (GLM) models and BIC model choice; Figure S1). Consequently, the remainder of the study focused on ${\text{IC}}_{\text{50}}$ and protein abundance. For more discussion on the biology of these traits, please see the Supplemental Information.

Having identified that epistatic interactions are likely to exist in the $IC_{50}$ and abundance traits (Figure 1 and Figure S1), we employed a set of regularized regressions to “decompose” the magnitudes, signs, and orders of epistatic effects operating at the different scales of genetic information represented in this data set (SNPs in DHFR associated with resistance to trimethoprim, species-specific amino acid background, and PQC mutations). Effect sizes can be found in Data S1 and S2.

### Decomposition of epistasis for ${\text{IC}}_{\text{50}}$

**Figure 2:**
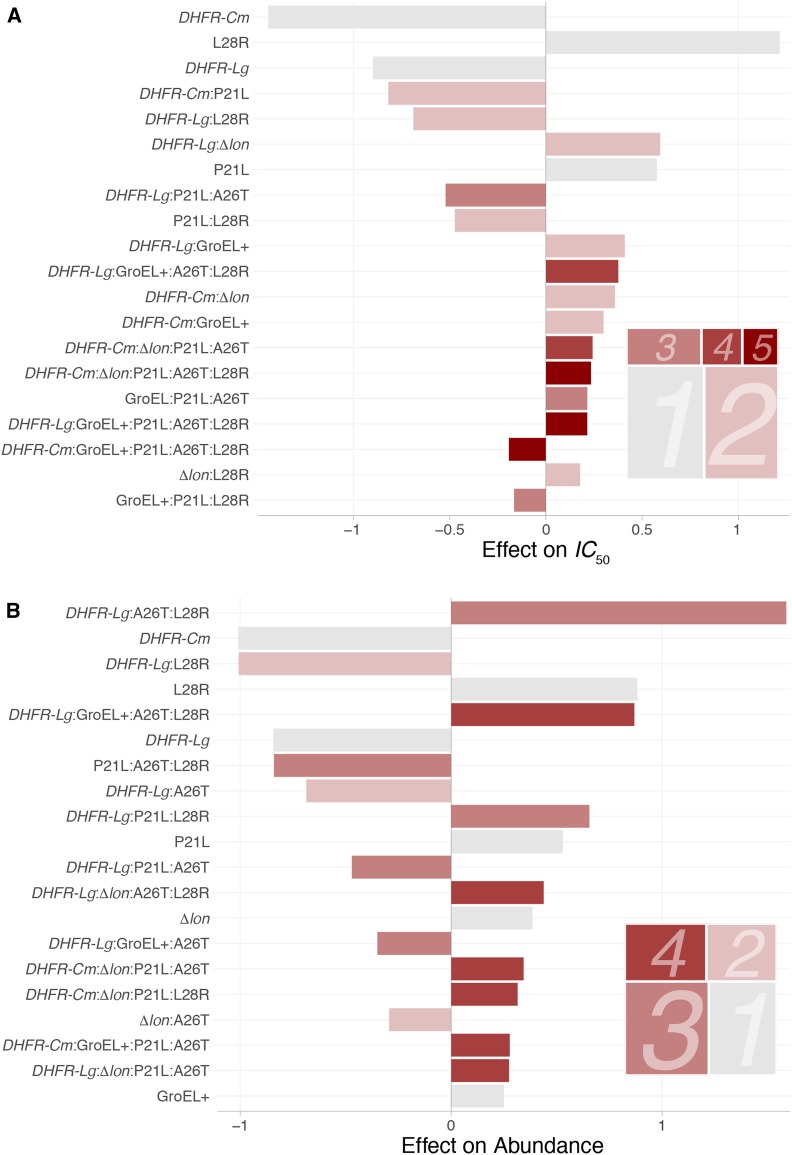
Widespread presence of higher-order interactions for (A)${\text{IC}}_{\text{50}}$ and (B) protein abundance. Distribution of regression coefficients from a LASSO regression allowing interactions across species, protein quality control context, and DHFR mutations for two phenotypes of interest. Bars represent the signs and magnitudes of the 20 largest coefficients in the best-fit model. Coefficients are arranged from top to bottom by their magnitude and their color represents their order (gray for main effects and increasing darkness of red for terms of order two through five). The treemaps in the bottom right corner of each panel represent the sum of all nonzero coefficients by order (the area of each box is the total effect of terms of that order). DHFR, dihydrofolate reductase; LASSO, least absolute shrinkage and selection operator.

The main driver of ${\text{IC}}_{\text{50}}$ is the species-specific amino acid background (Figure 2A). The *C. muridarum* and *L. grayi* amino acid backgrounds have the largest negative effects in the full LASSO regression for this trait (effect sizes −1.44 and −0.9, respectively). Taken alone, these findings suggest that the species DHFR context is an important factor in determining the ${\text{IC}}_{\text{50}}$ phenotype. Our knowledge of the biology of the system provides us with a mechanistically informed interpretation: prior studies have demonstrated that $DHFR_{Cm}$ is inefficient catalytically and that $DHFR_{Lg}$ is thermodynamically unstable (Rodrigues *et al.* 2016). Given that catalysis and thermostability are necessary for an enzyme to carry out its function, that the *C. muridarum* and *L. grayi* amino acid backgrounds have such strong negative effects on ${\text{IC}}_{\text{50}}$ is unsurprising. However, we cannot relegate the entirety of the main effects to species background: the second-largest effect overall is the presence of the L28R mutation (effect size = 1.22), demonstrating that main effect actors of various kinds can influence the ${\text{IC}}_{\text{50}}$ phenotype.

Even though main effects define the top three independent drivers of ${\text{IC}}_{\text{50}}$, higher-order interactions have a larger total effect than main effects on this trait (Figure 2A). Among interactions, the specific patterns are mechanistically diverse: some are between species-specific amino acid backgrounds and individual SNPs (*e.g.*, $DHFR_{Lg}$:L28R, effect size = −0.69), while others are between species-specific backgrounds and PQC environments ($DHFR_{Lg}$:GroEL+, effect size = 0.41). As with the main effects, several of these findings might be explained by our knowledge of the study system. Though there is a basis for the prediction that $DHFR_{Lg}$ and the GroEL+ phenotype would interact (the GroEL+ phenotype helps to stabilize the relatively unstable $DHFR_{Lg}$ enzyme), many of the calculated higher-order interactions cannot be so readily explained and might serve as the basis of future inquiry. Several plausible mechanistic interpretations are explored in Table 1.

### Decomposition of epistasis for protein abundance

As with the ${\text{IC}}_{\text{50}}$, Figure 2B shows that $DHFR_{Cm}$ has the strongest main effect on protein abundance. This reflects a general pattern of similarity in effects between ${\text{IC}}_{\text{50}}$ and abundance, which share their top three main effect factors: $DHFR_{Cm}$ (effect size = −1.01), L28R (effect size = 0.88), and $DHFR_{Lg}$ (effect size = −0.84). However, interactions appear to play a much larger role in determining protein abundance. We observe several notable patterns, with third-order interactions displaying the largest overall effect, defined by the interaction with the largest single effect (of any) on abundance: $DHFR_{Lg}$:A26T:L28R (effect size = 1.59). Conspicuously absent from the most important main effects are the PQC backgrounds (GroEL+ and Δ*lon*; effect sizes = 0.25 and 0.38, respectively). This suggests that PQC machinery is mostly a meaningful actor in determining DHFR abundance in the presence of other genetic parcels, or rather, only certain SNP and species background combinations seem to be significantly affected by the presence or absence of certain PQC variants. Table 2 proposes potential mechanisms that could explain several of these interactions, based on knowledge of the study system. As with ${\text{IC}}_{\text{50}}$, these proposed mechanisms are speculative, but could be the basis of more detailed inquiry in the future.

### ${\text{IC}}_{\text{50}}$ *vs.* abundance: correlation and pleiotropy

**Figure 3:**
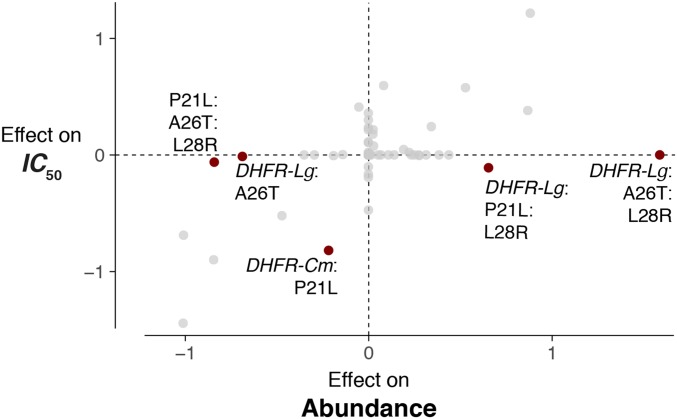
Epistatic effects are correlated between ${\text{IC}}_{\text{50}}$ and protein abundance traits, with several important higher-order outliers that demonstrate pleiotropic effects. Highlighted are the five terms with the largest discrepancies in value between the two phenotypes. DHFR, dihydrofolate reductase.

The determinants of ${\text{IC}}_{\text{50}}$ and protein abundance are similar, but there are meaningful and relevant outliers (Figure 3; $R^{2}=0.35$and GLM $p=10^{-7}$). The significant relationship between effect sizes estimated from our full models for ${\text{IC}}_{\text{50}}$ and abundance suggests that large-scale patterns of epistasis between these related traits are correlated. This correlation is not surprising: it reflects that these traits are connected at a mechanistic level, since bacteria need to make the enzyme to survive the effects of a drug that antagonizes that enzyme. More interesting are, perhaps, the outlier factors: the $DHFR_{Lg}$:A26T:L28R interaction has a strong effect on abundance (effect size = 1.59) and none on ${\text{IC}}_{\text{50}}$ (effect size = 0). Similarly, the $DHFR_{Lg}$:P21L:L28R interaction has a negative effect on ${\text{IC}}_{\text{50}}$ (effect size = −0.11) and a solidly positive effect on abundance (effect size = 0.66). Thus, at a more detailed level of analysis, we observe that individual effects can differ quite substantially, which highlights that certain mutation interactions can tune related phenotypes in different ways (in both magnitude and sign of effect). The differences in inferred effect sizes suggest that higher-order effects on abundance (P21:A26T:L28R, $DHFR_{Lg}$:P21L:L28R, and $DHFR_{Lg}$:A26T:L28R) need not translate into downstream effects on ${\text{IC}}_{\text{50}}$. In other words, we find in these differences some indication of pleiotropy, where mutations (or, in this case, interactions among mutations) display different effects on even functionally related phenotypes.

### Epistatic effects of SNPs across PQC contexts

**Figure 4:**
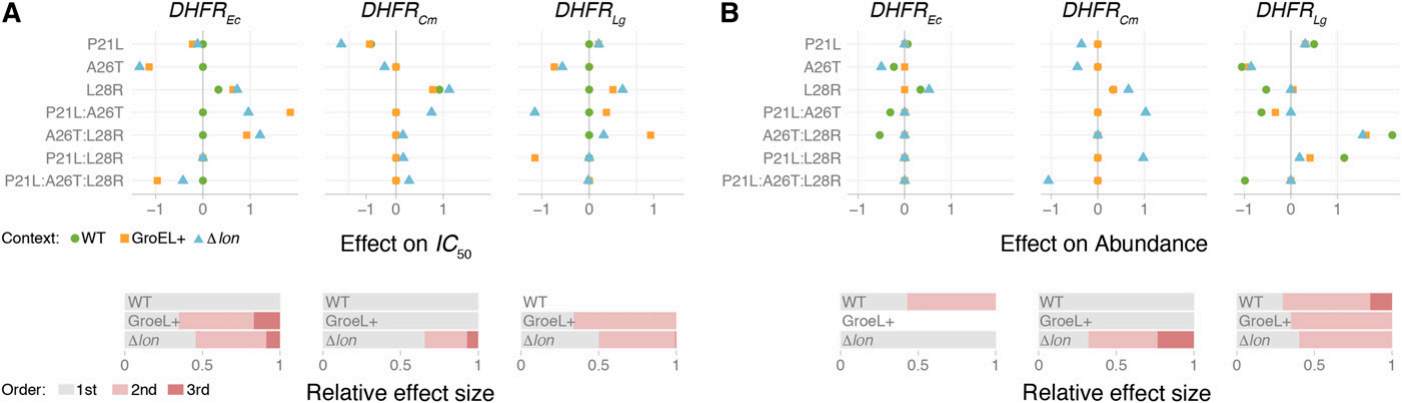
Magnitude, direction, and order of epistatic effects between SNPs across PQC-species backgrounds for (A) ${\text{IC}}_{\text{50}}$ and (B) DHFR abundance. The estimated effect of single-amino acid substitutions (P21L, A26T, and L28R) and their interactions vary across PQC backgrounds, indicating higher-order epistasis. Effect sizes were estimated using a LASSO regression within PQC-species background (see *Materials and Methods*). Dashed lines are drawn for clarity only. The bars at the bottom of each panel summarize the relative contribution of each order (main effects in gray, pair-wise interactions in light red, and third-order in dark red) to the total of (absolute) coefficients estimated in each model. DHFR, dihydrofolate reductase; LASSO, least absolute shrinkage and selection operator; PQC, protein quality control; WT, wild-type.

Having conducted analyses aimed at decomposing epistasis across the entire experimental data set (Figure 2 and Figure 3), we employed more granular methods to observe the phenotypic effects of the individual SNPs (P21L, A26T, and L28R) in various combinations relative to their putative ancestor (genotype 000 in each PQC-species group) as a function of PQC background. The coefficients, inferred by fitting nine separate LASSO models (one per PQC-species background), show considerable variation across PQC backgrounds and are consistent with the notion that PQC background is a direct modulator of epistatic effects. For ${\text{IC}}_{\text{50}}$, note the especially strong positive effects of the P21L:A26T ($DHFR_{Ec}$; effect size = 1.85) and the A26T:L28R ($DHFR_{Lg}$; effect size = 1.30) pairwise effects in the GroEL+ PQC context. The different PQC backgrounds have markedly different patterns of higher-order epistasis (Figure 4A), with Δ*lon* having notable pairwise interactions across SNP and species amino acid backgrounds.

For abundance, PQC background remains a powerful driver of epistatic effects, but in a manner much different from ${\text{IC}}_{\text{50}}$. In general, epistatic order differed substantially across PQC backgrounds (Figure 4B, bottom panel), with several especially notable effects in Δ*lon*: P21L:A26T and P21:L28R (both in $DHFR_{Cm}$; effect sizes = 1.03 and 0.98, respectively), and a third-order interaction P21L:A26T:L28R with a strongly negative effect (also in $DHFR_{Cm}$; effect size = −1.06).

## Discussion

In this study, we have attempted to dissect the epistatic interactions (in terms of magnitude, sign, and order) operating across SNPs, species-specific amino acid backgrounds, and PQC genetic backgrounds for two phenotypes related to drug resistance in bacteria. Below, we discuss the major findings, organized into several subsections. Additional discussion points can be found in the Supplemental Information.

### Higher-order epistatic interactions within and between genes influence two traits related to drug resistance

The results speak of the difficulty in making *a priori* assumptions about the way that epistasis operates when a system contains potential interactions of different kinds (*e.g.*, intragenic and intergenic). If we assume that physical distance between mutations correlates with the strength of interactions, we might guess that mutations within a gene (intragenic epistasis) might interact more readily than between genes (intergenic epistasis). However, this assumption is not supported by our results: we observed that higher-order interactions involving multiple SNPs and PQC backgrounds (*i.e.*, intergenic interactions) can have important effects on several phenotypes, often as large as intragenic interactions. Discussed in the light of modern evolutionary genetics, these results add further color to the debates surrounding the challenges of deconstructing complex phenotypes from effects of individual SNPs, as is often the goal of GWAS. For example, even in circumstances where we are successful in identifying SNPs that are significantly overrepresented in a population of individuals with a certain phenotype, interactions between these SNPs and any other unit of genetic information (perhaps outside of the gene where the candidate SNPs are located) may account very well for most of the variance in the phenotype of interest. That being the case, evolutionary geneticists are justified in being cautious in interpreting the importance of main effect SNPs on complex phenotypes.

### While epistasis patterns are correlated between related traits, several higher-order effects manifest uniquely across traits

Just as provocative as the observed epistatic interactions is the manner in which these factors influence related traits. Protein abundance affects how a microbe survives the presence of an antibiotic (trimethoprim in this case) through producing enough DHFR to perform the necessary catalytic functions. Protein abundance has been identified as a component of ${\text{IC}}_{\text{50}}$ in a quantitative approach used to predict the ${\text{IC}}_{\text{50}}$ from various biochemical and biophysical parameters (see Supplemental Information). Because of this, we would expect the patterns of epistasis between ${\text{IC}}_{\text{50}}$ and protein abundance to be well correlated (Figure 3). However, at another level of analysis, the nature and magnitude of individual effects are different between these traits: several higher-order effects that meaningfully influence protein abundance (both negatively and positively) have almost no effect on ${\text{IC}}_{\text{50}}$. We might summarize these findings another way: strong overall correlations between epistatic interactions acting on related traits still allow for meaningful differences in the identity and magnitude of individual interactions. When it comes to how certain epistatic interactions manifest, related traits might not be so related at all.

### Patterns of epistasis are broadly affected by PQC environments

We found candidate SNP interactions with large and specific effects on both ${\text{IC}}_{\text{50}}$ and abundance, but most differed across PQC backgrounds. Though the results in this study have further demonstrated how widespread epistasis can be, we have also identified how there are individual SNPs (or SNP combinations) that influence individual traits while having a minor influence on related ones. And so, despite the prevailing idea that epistasis undermines a simple answer to questions about how complex phenotypes are constructed, our effort to decompose the epistasis in this system has identified SNP/SNP interactions that could be summarized as being reliable signatures for the phenotypes measured in this study. However, these findings supplement recent studies that emphasize the importance of the recipient genome in understanding and predicting the phenotypic effects of transgenic mutations (Vogwill *et al.* 2016; Wang *et al.* 2016), as PQC context strongly dictated the consequences of these SNPs.

### Environmental influences on higher-order epistasis: moving toward mechanistic explanations

A simplistic summary of these results might suggest a conclusion along the lines of “epistasis implies that we can never fully decouple the heritable components of a complex trait” or “we can never predict the phenotypic consequences of a given SNP across different genotypic contexts.” These conclusions might be discouraging, especially to those who would prefer that main effects drive the phenotypes of interest (say, in a bioengineering setting). However, the data presented here are hardly the only results that would produce such disappointment, as complex traits without higher-order epistasis at work are quickly becoming the exception. That epistasis produces spurious phenotypic effects is an unambiguous theme of the results of this study (reflected most directly in Figure 2 and Figure 4), supporting recent studies that affirm the presence of higher-order epistasis across a wide breadth of phenotypes, in many organisms.

Moreover, we argue that such broad summaries of epistasis patterns are unnecessary, as our analysis allows us to discuss epistasis at a greater (and more useful) level of detail. We specifically demonstrate how individual components of a critical physiological determinant (PQC environment) shape how epistasis manifests in a single protein, across two phenotypes. Note that, in prior studies, GroEL+ and Δ*lon* were demonstrated to have similar effects on DHFR mutations. In this study, their respective cytoplasmic environments shaped higher-order interactions differently (whatever the magnitude) across different traits. For example, the results suggest where to start if we ever wanted to tune the phenotypes in this study in a certain direction. We found that the L28R main effect has a positive influence on ${\text{IC}}_{\text{50}}$ in many contexts, and that the A26T:L28R combination powerfully influences DHFR abundance in the *L. grayi* background.

Lastly, our approach does more than simply resolve how epistatic interactions drive a set of phenotypes. These results also offer a small step toward what might be the future of the study of epistasis, where statistical methods reveal potential mechanisms or generate testable hypotheses for how parcels of genetic information interact in constructing complex phenotypes. This perspective will be necessary if true genetic modification (as driven by clustered regularly interspaced short palindromic repeats or other methods) will ever become commonplace. Eventually, we will need to know what to expect when we engineer a given mutation into a given background: how that mutation interacts with others (across the genome), how we might finely tune such interactions, or if we should bother trying at all.
